# Commentary: Understanding the Appropriate and Beneficial Use of Before and After Photos in Breast Surgery: A North American Survey

**DOI:** 10.1177/22925503231172798

**Published:** 2023-05-01

**Authors:** Tyler Safran, Joshua Vorstenbosch

**Affiliations:** Division of Plastic & Reconstructive Surgery, 54473 McGill University Health Centre, Montreal, Quebec, Canada

Before and after photos dominate the plastic surgery social media landscape and play a major role in breast surgery.^1^ Despite their ubiquity, we know very little about how patients interested in pursuing or who have recently had breast surgery perceive before and after photos. In this article, Datta et al^2^ use the MTurk crowd-sourcing tool to survey individuals pursuing or who have had breast surgery to ascertain the aspects of before and after photos that patients deem important. The 72 responses recorded in this observational study demonstrate that patients believe that before and after photos are important components of the informed consent process that should be shown via private links or in surgeons’ offices and illustrate a broad spectrum of body types and time elapsed from surgery.

This study confirms that patients utilize before and after photographs to help decide on a surgeon and type of surgery, with 92% having found and used relatable before and after photos when deciding on a surgeon. The authors also show that patients prefer unedited and standardized images, different time points, and close-ups of scars. Interestingly, although participants wanted to see different possible outcomes and scarring, they did not seek photos of complications. By contrast, if patients were shown possible complications, would they be as interested in pursuing more extreme surgeries that might result in a higher rate of complications? Or would patients still be tempted by certain surgeries if they saw pictures of the possible complications? Perhaps the patient disinterest in photographs of complications demonstrates the importance of showing them during the informed consent process. For example, a patient with a family history of keloid scarring might benefit from seeing pictures of a wise-pattern mastopexy complicated by keloid scarring as opposed to the faint linear scaring seen on the photo posted to social media that is intentionally placed to elicit a positive response from prospective patients.

Several studies, including one by the authors of this article, explore surgeons’ perspectives on before and after photos of plastic surgery.^3^ A strength of this study is that the authors build on their previous results from the “Nominal Group Technique” to identify the intersection of priorities that surgeons and patients assign to before and after photos. Investigations such as this represent important incremental steps toward standardization of the use of before and after photos in plastic surgery to meet the goals of surgeons and patients alike. This study overall was well thought out, with the only major weaknesses lying in the inherent limitations of the MTurk program and a somewhat low number of responses. However, the authors propose important next steps including a nationwide Delphi analysis to establish the “appropriate” and “beneficial” use of before and after photos in breast surgery.

We congratulate the authors of this article on identifying patient perspectives on the use of before and after photos in breast surgery. This manuscript represents an important step towards more standardized use of before and after photos, and we look forward to reading more of the group's future work on this topic.
